# Global methylation in relation to methotrexate-induced oral mucositis in children with acute lymphoblastic leukemia

**DOI:** 10.1371/journal.pone.0199574

**Published:** 2018-07-09

**Authors:** Natanja Oosterom, Pieter H. Griffioen, Marissa A. H. den Hoed, Rob Pieters, Robert de Jonge, Wim J. E. Tissing, Marry M. van den Heuvel-Eibrink, Sandra G. Heil

**Affiliations:** 1 Princess Máxima Center for Pediatric Oncology, Utrecht, The Netherlands; 2 Department of Clinical Chemistry, Erasmus MC University Medical Center, Rotterdam, The Netherlands; 3 Department of Pediatric Oncology/Hematology, Erasmus MC University Medical Center-Sophia’s Children’s Hospital, Rotterdam, The Netherlands; 4 Department of Clinical Chemistry, VU Medical Center, Amsterdam, The Netherlands; 5 Department of Clinical Chemistry, Amsterdam Medical Center, Amsterdam, The Netherlands; 6 Department of Pediatric Oncology, University of Groningen, University Medical Center Groningen, Beatrix Children’s Hospital, Groningen, The Netherlands; RWTH Aachen University Medical School, GERMANY

## Abstract

**Background:**

Children with acute lymphoblastic leukemia (ALL) often suffer from toxicity of chemotherapeutic drugs such as Methotrexate (MTX). Previously, we reported that 20% of patients receiving high-dose MTX developed oral mucositis. MTX inhibits folate metabolism, which is essential for DNA methylation. We hypothesize that MTX inhibits DNA methylation, which results into adverse effects. We studied DNA methylation markers during high-dose methotrexate treatment in pediatric acute lymphoblastic leukemia (ALL) in relation to developing oral mucositis.

**Materials & methods:**

S-Adenosyl-Methionine (SAM) and S-Adenosyl-Homocysteine (SAH) levels and LINE1 DNA methylation were measured prospectively before and after high-dose methotrexate (HD-MTX 4 x 5g/m2) therapy in 82 children with ALL. Methotrexate-induced oral mucositis was registered prospectively. Oral mucositis (grade ≥ 3 National Cancer Institute Criteria) was used as clinical endpoint.

**Results:**

SAM levels decreased significantly during methotrexate therapy (-16.1 nmol/L (-144.0 –+46.0), p<0.001), while SAH levels and the SAM:SAH ratio did not change significantly. LINE1 DNA methylation (+1.4% (-1.1 –+6.5), p<0.001) increased during therapy. SAM and SAH levels were not correlated to LINE1 DNA methylation status. No association was found between DNA methylation markers and developing oral mucositis.

**Conclusions:**

This was the first study that assessed DNA methylation in relation to MTX-induced oral mucositis in children with ALL. Although global methylation markers did change during methotrexate therapy, methylation status was not associated with developing oral mucositis.

## Introduction

Treatment outcome of pediatric acute lymphoblastic leukemia (ALL) has improved substantially over the past decades, with 5-year survival rates currently reaching 90% in developed countries [1–3]. We previously showed that 20% of children with ALL receiving high-dose MTX developed oral mucositis as an adverse effect despite folate rescue therapy [4]. It would be of clinical value to identify predictors of MTX-induced oral mucositis to select patients who could benefit from personalized intervention strategies [2].

**Fig 1:**
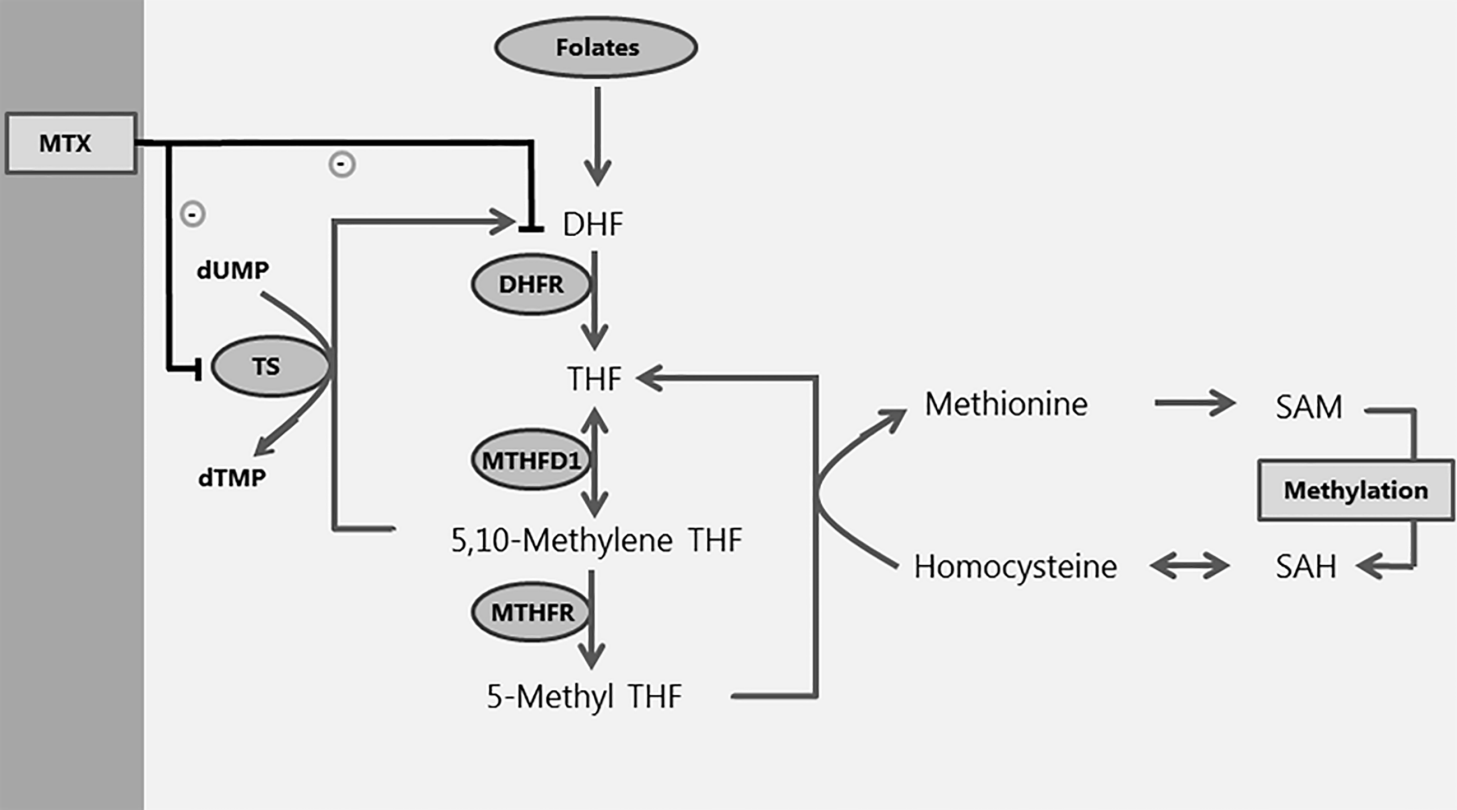
Role of MTX in relation to one-carbon metabolism. SAM: S-adenosylmethionine; SAH: S-adenosylhomocysteine; DHF: dihydrofolate; THF: tetrahydrofolate; TS: thymidylate synthase; DHFR: dihydrofolate reductase; MTHFD1: methylenetetrahydrofolate dehydrogenase 1; MTHFR: methylenetetrahydrofolate reductase; MTX: methotrexate. Folic acid donates a methyl-group to the one-carbon metabolism pathway. Through several steps methionine is transformed into SAM, which then donates the methyl-group for the DNA methylation process, and is transformed into SAH and homocysteine. MTX inhibits DHFR and TS. By inhibiting DHFR, MTX inhibits the pathway leading to methylation.

MTX is an inhibitor of methionine-adenosine transferase (MAT), resulting in lower levels of methionine with concomitant lower S-adenosyl-methionine (SAM) and is therefore expected to decrease DNA methylation [5, 6]. In a mouse neural tube defect model, MTX caused DNA hypomethylation [7]. In contrast, low-dose methotrexate caused global DNA hypermethylation in rheumatoid arthritis patients [8, 9]. DNA methylation is the process in which methyl-groups (-CH_3_) bind to Cytosine-phosphate-Guanine dinucleotides (CpG) in the DNA, by which it plays a role in ‘gene-silencing’ [10]. Methyl-groups are obtained from one-carbon metabolism, during which the methyl-group from SAM is donated to DNA, RNA and proteins, after which S-adenosyl-homocysteine (SAH) is formed (Fig 1). Plasma SAM and SAH metabolite levels and the SAM:SAH ratio reflect the global intracellular methylation status of the cell. Disturbances in SAM—SAH levels in combination with a decreased SAM:SAH ratio are associated with DNA hypomethylation [11]. Global DNA methylation status can be quantified by several methods, amongst which measuring DNA methylation status of Long Interspersed Nuclear 1 elements (*LINE1*). *LINE1* elements occur frequently (~20.000 copies) in the human genome and DNA methylation status of these elements is therefore considered to be a proxy for global DNA methylation [12].

Currently, no studies on changes in DNA methylation in relation to the development of chemotherapy-related oral mucositis exist. However, DNA methylation has been implicated as a possible biomarker of treatment-related toxicity in other malignancies and rheumathoid arthritis treatment [13–16].

In the current prospective study we explored the hypothesis, that high-dose MTX therapy inhibits global DNA methylation, which is associated with the development of MTX-induced oral mucositis in children with ALL.

## Materials & methods

### Patients, treatment protocol and toxicity evaluation

The patient cohort and treatment protocol have been previously reported [4]. Briefly, patients between 1 and 18 years treated according to the standard and medium risk arms of the Dutch Childhood Oncology ALL-10 protocol (2004–2012) were eligible for the current study [17]. The study was approved by the Medical Ethical Committee (MEC-2005-358). Written informed consent was obtained before data- and sample collection. An overview of protocol M (HD-MTX phase; 5 gram/m^2^/course) is shown in S1 Fig. A modified version of The National Cancer Institute (NCI) Common Terminology Criteria for Adverse Events v.3.0 score system was used to score and document toxicity (S1 Table) [18]. Clinically relevant oral mucositis, defined as NCI grade ≥ 3, was used as endpoint in the analyses [4]. The highest grade of toxicity observed in each patient during protocol M was documented.

### Sample collection

Peripheral EDTA blood samples were collected before the first HD-MTX course (T0) as well as two weeks after discontinuation of protocol M (T1) and were stored at -80°C (S1 Fig) [17]. DNA was isolated from whole blood using the MagNA Pure Compact Nucleid Acid isolation kit (Roche Molecular Biochemicals^®^) according to the manufacturer's instructions.

### Cellular- and global DNA-methylation status

We measured plasma SAM and SAH levels as a proxy for cellular methylation status and *LINE1* DNA methylation as a proxy for global DNA methylation status at T0 and T1.

SAM and SAH plasma levels were measured using a liquid chromatography tandem-mass spectrometry (LC-MS/MS) method using solid-phase extraction columns as previously described [19]. The SAM:SAH ratio was calculated. The *LINE1* global DNA methylation assay was performed using primers as previously reported [20]. The *LINE1* assay measured the methylation percentage at 12 CpG sites. Primers were designed using EpiTYPER Designer software (http://www.epidesigner.com/). We used a primer melting temperature of 64 °C, a primer size of 25 bp and an amplicon length of 300 bp. Primer sequences are depicted in S2 Table.

For global *LINE1* DNA methylation assay, isolated DNA (500 ng) was treated with sodium bisulphite to discriminate between methylated and unmethylated cytosines using the EZ DNA Methylation^™^ Kit (Zymo Research^®^) according to the manufacturer's instructions. Bisulphite-treated DNA was stored at +4°C and processed within 1 week according to the manufacturer’s instructions. The assay was performed in triplets per patient at T0 and T1 and mean DNA methylation values were calculated from these triplets when the variation coefficient was <10%. A PCR to amplify bisulphite-treated DNA was performed using the C-1000 Touch Thermal Cycler^™^ (Bio-Rad). Two μl of sodium bisulphite-treated DNA was added to each reaction (total volume reaction: 12 μl). The PCR master mix consisted of 1.2 μl 10x Buffer, 1.2 μl 2mM deoxyribonucleotide triphosphates (dNTPs), 0.7 μl 25mM MgCl_2_, 2 μl of the forward and reverse primer (1 pmol/μl), 0.1 μl AmpliTaq (5 *U/L*, Applied Biosystems, Waltham, MA, USA) and 2.8 μl H_2_O. A standard ‘step-down’ PCR thermal cycling protocol was performed: 10 minutes at 95°C; 5 cycles of 20 seconds (s) at 95°C, 30 s at 65°C and 1 minute at 72°C; 5 cycles of 20 s at 95°C, 30 s at 58°C and 1 minute at 72°C; 39 cycles of 20 s at 95°C, 30 s at 53°C, 1 minute at 72°C and a final elongation step for 3 min at 72°C followed by infinite hold at 12°C. After the PCR, a dilution of 10 μl H_2_O, 3 μl PCR product and 2 μl loading dye was loaded onto a 2% agarose gel to verify whether the PCR was successful. Unincorporated PCR primers and deoxynucleotide triphosphates in the samples were inactivated by using shrimp alkaline phosphatase (SAP) treatment as previously described [21]. Reverse transcription/RNase T cleavage was performed using the following conditions: 3.21 μl of RNase-free double-distilled H_2_O (ddH_2_O), 0.89 μl of 5x T7 polymerase buffer, 0.22 μl T Cleavage mix, 2.2 mM DTT, 20 *U* T7 DNA & RNA polymerase and 0.6 μg RNase A in 2 μl of purified DNA product. This mixture was incubated at 37°C for 3 hours. Thereafter, 6 mg of Clean Resin and 20 μl of milliQ H_2_O were added to each sample, rotated slowly for 20 minutes to mix reagents and then centrifuged down for 5 minutes at 3000rpm. Thereafter, 10–15 nL of the cleavage reaction was dispensed onto a SpectroCHIP array with a Nanodispenser RS1000 (Sequenom). The array was performed on a Matrix-assisted Laser Desorption/Ionization—Time Of Flight (MALDI-TOF) MassARRAY (Sequenom) analyzer according to the manufacturer’s instructions. LINE1 CpG4 could not be measured due to a silent signal. LINE1 CpG10 could not be analyzed due to a low mass fragment. LINE1 CpG6 and 7, CpG8 and 9 and CpG11 and 12 were each analyzed together as the two CpG sites were present in one fragment. A precision experiment was performed consisting of measuring 10 DNA controls, which should give DNA methylation percentages with a variation coefficient of <10%.

### Statistical analysis

Statistical analyses were performed using SPSS Statistics Version 20.0.0.1 (SPSS, Chicago, IL, USA). For the *LINE1* global DNA methylation assay the methylation percentage of individual CpG sites and the mean methylation percentage of all CpG sites measured in the assays was used for statistical analysis. Changes in SAM—SAH levels and the global *LINE1* DNA methylation status between T0 and T1 were tested using a paired T test (mean ± standard deviation) or a Wilcoxon Rank Sum test (median, range), as appropriate, based on the normal distribution of data. The association between SAM—SAH levels and global *LINE1* DNA methylation status at T0 and the change between T0 and T1 (delta T1 –T0) with the development of MTX-induced oral mucositis was tested using an independent T test (mean ± standard deviation) or a Mann Whitney U test (median, range) as appropriate. The correlation between SAM—SAH levels and *LINE1* DNA methylation was tested using a Spearman’s rho coefficient. A correlation coefficient of >0.7 was considered relevant. In view of multiple comparisons the significance level was set at a p-value of 0.004 using a Bonferroni correction (p-value = 0.05 / 12). We tested the possible confounding effect of clinical characteristics (age at diagnosis, gender, ALL immunophenotype, ALL risk group) by testing whether these factors were significantly (p <0.05) related to both the determinant (DNA methylation) and the outcome (mucositis). If confounders were significant in both these univariate analyses, they were included in a multivariate regression model.

## Results

Plasma SAM—SAH levels and *LINE1* DNA methylation were measured before start of MTX therapy (T0) and two weeks after end of MTX therapy (T1) in 82 pediatric ALL patients (Table 1). *LINE1* methylation percentages were measured at all CpG sites with a variation coefficient of <10%. In total, 17 patients (21%) patients developed MTX-induced oral mucositis ≥ NCI grade 3. Baseline characteristics are summarized in Table 1.

**Table 1:** Baseline characteristics (n = 82).

Patient characteristics	
**Age at diagnosis**	
**median (range in years)**	5.4 (1–18)
**Sex, n (%)**	
**Female**	46 (56)
**Male**	36 (44)
**Immunophenotype ALL, n (%)**	
**B-lineage**	71 (87)
**T-lineage**	11 (13)
**Risk group ALL-10 protocol, n(%)**	
**Standard risk**	23 (28)
**Medium risk**	59 (72)
Mucositis, n (%)*	
**No**	65 (79)
**Yes**	17 (21)

*Clinically relevant mucositis is defined as ≥ grade 3 according to the National Cancer Criteria v.3.0. [18].

### Methylation markers—Changes during MTX therapy

Methylation marker levels at T0 and T1 are described in Table 2. Plasma SAM levels were significantly (mean -16.1 nmol/L [-144.0 –+46.0]) lower after MTX therapy (p-value < 0.001), whereas SAH levels and SAM:SAH ratio did not change significantly (Table 2). *LINE1* DNA methylation increased (mean +1.4% [-1.1 –+6.5]) during MTX therapy (p-value <0.001, Table 2 + S3 Table). SAM—SAH levels and *LINE1* DNA methylation status at T0 and T1 were not correlated (S4 Table).

**Table 2:** Methylation before and after stop of MTX therapy.

		T0 (before start MTX)	T1 (after stop MTX)	p-value
**Cellular methylation**				
SAM (nmol/L), median (range)	(n = 77)	109.0 (71.0–245.0)	99.0 (44.0–151.0)	<0.001*
SAH (nmol/L), median (range)	(n = 77)	13.5 (8.1–78.2)	12.9 (6.4–56.2)	0.234
SAM:SAH ratio, mean ± SD	(n = 77)	8.0 ± 2.8	7.4 ± 3.1	0.207
**Global DNA methylation**				
LINE1 –methylation (%), mean ± SD	(n = 80)	65.1 ± 1.8	66.5 ± 1.9	<0.001*

T0: before start MTX; T1: after stop MTX. Mean percentage methylation of CpG sites in LINE1 (%) and plasma SAM—SAH levels (nmol/L) at T0 vs. T1; mean ± SD or median (range) based on normal distribution of data.

### Methylation status in relation to MTX-induced oral mucositis

SAM and SAH levels and the SAM:SAH ratio at T0 were not associated with the occurrence of MTX-induced oral mucositis (Table 3). *LINE1* DNA methylation at T0 was not significantly associated with the development of MTX-induced oral mucositis (Table 3 + S5 Table). In addition, changes in the methylation markers between T0 and T1 were not significantly associated with the development of MTX-induced oral mucositis (Table 3 + S6 Table). None of the tested clinical confounders (age at diagnosis, gender, ALL immunophenotype, ALL risk group) significantly affected these analyses.

**Table 3:** SAM and SAH levels and LINE1 DNA methylation in relation to MTX-induced oral mucositis.

*T0*			*p-value*	*Change T0-T1*			*p-value*
Cellular methylation							
**SAM (nmol/L), median (range)**				**SAM (nmol/L), median (range)**			
No Mucositis	n = 62 (78)	109.5 (72.0–245.0)	0.788	No Mucositis	n = 62 (81)	-12.5 (-144.0 –+46.0)	0.338
Mucositis	n = 17 (22)	107.0 (71.0–151.0)		Mucositis	n = 15 (19)	-9.0 (-46.0 –+31.0)	
**SAH (nmol/L), median (range)**				**SAH (nmol/L), median (range)**			
No Mucositis	n = 62 (78)	13.7 (8.1–58.8)	0.407	No Mucositis	n = 62 (81)	-1.0 (-33.8 –+46.0)	0.979
Mucositis	n = 17 (22)	11.4 (6.3–78.2)		Mucositis	n = 15 (19)	0.4 (-53.6 –+11.1)	
**SAM:SAH ratio, mean ± SD**				**SAM:SAH ratio, mean ± SD**			
No Mucositis	n = 62 (78)	7.9 ± 2.8	0.405	No Mucositis	n = 62 (81)	-0.7 **±** 3.6	0.501
Mucositis	n = 17 (22)	8.6 ± 3.1		Mucositis	n = 15 (19)	0.0 **±** 3.3	
Global DNA methylation							
**LINE1 –total methylation (%), mean *± SD***				**LINE1 –total methylation (%), mean ± SD**			
No Mucositis	n = 65 (79)	65.0 (± 1.9)	0.339	No Mucositis	n = 63 (79)	1.5 ± 1.4	0.290
Mucositis	n = 17 (21)	65.5 (± 1.3)		Mucositis	n = 17 (21)	1.1 ± 1.2	

Mean percentage methylation of LINE1 and plasma SAM—SAH levels in nmol/L before start of MTX (T0) and the change (T0 –T1) during MTX therapy in relation to MTX-induced oral mucositis; mean ± SD or median based on normal distribution of data.

## Discussion

This is the first study on the role of cellular methylation status and global DNA methylation in relation to the development of MTX-induced oral mucositis in children with ALL. Although we showed that global DNA methylation markers were changed after MTX therapy, plasma SAM—SAH levels and *LINE1* DNA methylation were not associated with developing oral mucositis due to high-dose MTX treatment.

We observed a decrease in SAM levels after high-dose MTX treatment in pediatric ALL patients, while the SAH levels and the SAM:SAH ratio did not show a significant change. This very likely means that the change in SAM was not large enough to affect the SAM:SAH ratio significantly. Inhibition of SAM levels has been shown previously in an in vitro methotrexate model and a methotrexate-induced neural tube defect mouse model [7, 22]. Our study in the pediatric ALL setting using a high-dose MTX regimen confirmed these results. However, this decrease in SAM seen at the end of MTX therapy can also be caused by other factors, such as environmental factors. More studies are necessary to assess the role of MTX in relation to plasma SAM.

We observed small increases of 1–2% in *LINE1* DNA methylation status after high-dose MTX treatment, which are are in line with previous reports in other diseases [9, 23]. For example, in patients using selective serotonin reuptake inhibitors, global and gene-specific methylation differences of 1–5% were found to be associated with treatment response [23]. Furthermore, 5-methylcytosine levels, which is another global DNA methylation measure, differed between healthy controls and rheumathoid arthritis patients receiving MTX with 1–2% [9]. The observed increase in DNA methylation status after HD-MTX therapy should be replicated in an independent case-control setting or validated using another global DNA methylation assay than the LINE1 assay to assess whether the observed changes are due to HD-MTX and not due to other environmental factors, such as nutrition. The *LINE1* hypermethylation we found was contradictory to what we hypothesized, as MTX is expected to inhibit DNA methylation. In line with our results, previous studies in rheumathoid arthritis patients showed that MTX treatment induced DNA hypermethylation [8, 9].

A possible explanation for the observed DNA hypermethylation after MTX therapy in our study is that concomitant therapy is administered, such as folate rescue therapy and 6-Mercaptopurine. Folates provide methyl-groups necessary for methylation reactions and increased DNA methylation status in several mouse tissues (liver, kidney, brain) as well as in peripheral mononuclear cells [24, 25]. In contrast, 6-Mercaptopurine causes DNA hypomethylation [26]. It is possible that the inhibitory effect of MTX and 6-Mercaptopurine on DNA methylation status is masked by folate rescue therapy, as folate increases DNA methylation status.

Our study showed no association between global methylation markers at start and at the end of MTX therapy in relation to MTX-induced oral mucositis. These results do not confirm the hypothesis that global methylation is associated with MTX-induced mucositis. However, a recent study showed that the hypermethylation in CpG1 and CpG2 of the promotor of the ɣ-Glutamyl Hydrolase (GGH) gene, which is involved in polyglutamating MTX, can significantly reduce GGH mRNA expression in pediatric ALL [27]. Therefore, performing genome-wide DNA methylation analyses using an Illumina methylation EPIC array could be relevant in future studies to assess whether gene-specific DNA methylation status could be used as a possible biomarker in predicting MTX-induced toxicity, such as oral mucositis.

Finally, in our study DNA methylation status was measured in DNA isolated from whole blood leucocytes. At this point in therapy, patients are considered to be in complete remission, and therefore the DNA methylation profile of leukemic blasts, which are known to be different from normal leucocytes, should not interfere with our analysis. DNA methylation status differs per tissue [28, 29]. In future studies, it would be interesting to look into DNA methylation changes in the oral mucosa in relation to MTX-induced oral mucositis.

Strengths of our study are the prospective collection of toxicity data and the fact that all patients were treated according to the same standardized treatment protocol. A limitation is the relatively small sample size of this study.

## Conclusion

This study is the first report to study methylation status in relation to the development of MTX-induced oral mucositis in children with ALL. Global methylation markers changed after high-dose MTX therapy, but we could not demonstrate that global methylation markers are associated with the development of MTX-induced oral mucositis in pediatric ALL.

## Supporting information

S1 Fig Overview protocol M. Protocol M consists of a 57-day period in which patients receive 6-Mercaptopurine (6-MP) orally in a dose of 25 mg/m^2^/day. Patients receive 2-weekly high-dose methotrexate (HD-MTX) intravenously in a dose of 5000 mg/m^2^/dose in 24 hours. Intrathecal infusions of methotrexate, cytarabine (ARA-C) and di-adreson F (DAF) are administered 2-weekly. Leucovorin is administered at 36 hours, 42 hours and 48 hours after start of the HD-MTX infusion at a dose of 15 mg/m^2^/dose. Peripheral EDTA blood samples were collected at day 1 of protocol M (T0)) as well as two weeks after discontinuation of protocol M (T1).
(TIF)

S1 Table National Cancer Institute (NCI) Criteria oral mucositis. (DOCX)

S2 Table Sequences of forward (F) and reverse (R) primers in bisulphite treated DNA. (DOCX)

S3 Table LINE1 DNA methylation status (percentage) at T0 and T1 per single CpG site. (DOCX)

S4 Table Correlation coefficients SAM, SAH, SAM/SAH ratio and LINE1. (DOCX)

S5 Table LINE1 DNA methylation status (percentage) at T0 in relation to MTX-induced oral mucositis per single CpG site. (DOCX)

S6 Table Change in LINE1 DNA methylation status (Delta T1 –T0 percentage) during MTX therapy in relation to MTX-induced oral mucositis per single CpG site. (DOCX)

## Abbreviations

ALL: Acute Lymphoblastic Leukemia
CpG: Cytosine-phosphate-Guanine dinucleotide
LINE1: Long Interspersed Nuclear Elements 1
MAT: Methionine Adenosine Transferase
MTX: Methotrexate
NCI: National Cancer Institute
SAH: S-adenosylhomocysteine
SAM: S-adenosylmethionine
SAP: Shrimp Alkaline Phosphatase
