# Supplementary figures and images for: Global methylation in relation to methotrexate-induced oral mucositis in children with acute lymphoblastic leukemia
Source: PLoS One. 2018 Jul 9;13(7):e0199574. doi: 10.1371/journal.pone.0199574 (PMC6037363; doi:10.1371/journal.pone.0199574)

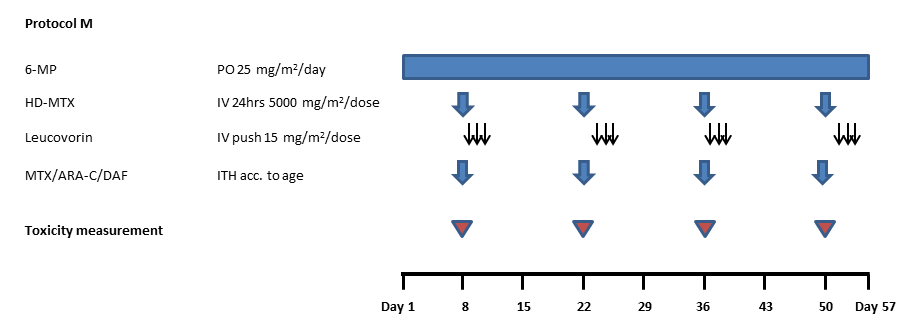

Supplement: S1 Fig — Protocol M consists of a 57-day period in which patients receive 6-Mercaptopurine (6-MP) orally in a dose of 25 mg/m2/day. Patients receive 2-weekly high-dose methotrexate (HD-MTX) intravenously in a dose of 5000 mg/m2/dose in 24 hours. Intrathecal infusions of methotrexate, cytarabine (ARA-C) and di-adreson F (DAF) are administered 2-weekly. Leucovorin is administered at 36 hours, 42 hours and 48 hours after start of the HD-MTX infusion at a dose of 15 mg/m2/dose. Peripheral EDTA blood samples were collected at day 1 of protocol M (T0)) as well as two weeks after discontinuation of protocol M (T1). (TIF) [file pone.0199574.s001.tif]
