# Supplementary material for: Global methylation in relation to methotrexate-induced oral mucositis in children with acute lymphoblastic leukemia
Source: PLoS One. 2018 Jul 9;13(7):e0199574. doi: 10.1371/journal.pone.0199574 (PMC6037363; doi:10.1371/journal.pone.0199574)
Supplement: S1 Table — (DOCX) [file pone.0199574.s002.docx]

***Supplemental Table 1: National Cancer Institute (NCI) Criteria oral mucositis***

| Adverse event  Grade | *0* | *1* | *2* | *3* | *4* | *5* |
| --- | --- | --- | --- | --- | --- | --- |
| Mucositis/ stomatitis of the oral cavity | Normal | Erythema of the mucosa. Minimal symptoms, normal diet. | Patchy ulcerations, symptomatic but can eat and swallow modified diet | Confluent ulcerations, bleeding with minor trauma. Symptomatic and unable to adequately aliment or hydrate orally. | Tissue necrosis, significant spontaneous bleeding. Symptoms associated with life-threatening consequences | Death |

*Oral mucositis ≥ NCI grade 3 was considered as clinically relevant endpoint in this study.*
