# Supplementary material for: Global methylation in relation to methotrexate-induced oral mucositis in children with acute lymphoblastic leukemia
Source: PLoS One. 2018 Jul 9;13(7):e0199574. doi: 10.1371/journal.pone.0199574 (PMC6037363; doi:10.1371/journal.pone.0199574)
Supplement: S2 Table — (DOCX) [file pone.0199574.s003.docx]

***Supplemental Table 2: sequences of forward (F) and reverse (R) primers in bisulphite treated DNA***

| ***Assay*** | ***Primer sequence*** |
| --- | --- |
| LINE1 | F: aggaagagagGTGTGAGGTGTTAGTGTGTTTTGTT  R: cagtaatacgactcactatagggagaaggctATATCCCACACCTAACTCAAAAAAT |
