# Supplementary material for: Global methylation in relation to methotrexate-induced oral mucositis in children with acute lymphoblastic leukemia
Source: PLoS One. 2018 Jul 9;13(7):e0199574. doi: 10.1371/journal.pone.0199574 (PMC6037363; doi:10.1371/journal.pone.0199574)
Supplement: S3 Table — (DOCX) [file pone.0199574.s004.docx]

***Supplemental Table 3. LINE1 DNA methylation status (percentage) at T0 and T1 per single CpG site***

| Global *LINE1* DNA methylation |  | T0 % | T1  % | p-value |
| --- | --- | --- | --- | --- |
| LINE1 - CpG1, mean ± SD | (n = 78) | 64.9 ± 3.8 | 68.7 ± 3.2 | **<0.001*** |
| LINE1 – CpG2, mean ± SD | (n = 80) | 59.4 ± 1.8 | 60.7 ± 1.6 | **<0.001*** |
| LINE1 - CpG3, mean ± SD | (n = 82) | 70.7 ± 2.0 | 70.1 ± 11.3 | 0.638 |
| LINE1 - CpG5, mean ± SD | (n = 78) | 38.9 ± 1.5 | 39.3 ± 1.5 | **0.003*** |
| LINE1 - CpG6.7, mean ± SD | (n = 74) | 70.6 ± 2.6 | 69.2 ± 11.8 | 0.342 |
| LINE1 - CpG8.9, median (range) | (n = 80) | 68.0 (58.5 – 71.3) | 68.7 (61.0 – 73.3) | **<0.001*** |
| LINE1 - CpG11.12, median (range) | (n = 82) | 84.0 (77.0 – 86.3) | 84.3 (83.6 – 85.3) | **<0.001*** |

*T0: before start MTX; T1: after stop MTX. Percentage methylation of individual CpG sites in LINE1 (%) at T0 vs. T1; mean ± SD or median (range) based on normal distribution of data*
