# Supplementary material for: Global methylation in relation to methotrexate-induced oral mucositis in children with acute lymphoblastic leukemia
Source: PLoS One. 2018 Jul 9;13(7):e0199574. doi: 10.1371/journal.pone.0199574 (PMC6037363; doi:10.1371/journal.pone.0199574)
Supplement: S4 Table — (DOCX) [file pone.0199574.s005.docx]

***Legend Supplemental Table 4. Correlation coefficients SAM, SAH, SAM/SAH ratio and LINE1***

|  | LINE1 total methylation T0 | | | Delta LINE1 total methylation T1-T0 |  |
| --- | --- | --- | --- | --- | --- |
| SAM (nmol/L) T0 | Correlation Coefficient | -,042 | 0.157 | | |
|  | Sig. (2-tailed) | ,713 | 0.173 | | |
|  | N | 79 | 77 | | |
| SAH (nmol/L) T0 | Correlation Coefficient | -,134 | 0.103 | | |
|  | Sig. (2-tailed) | ,239 | 0.372 | | |
|  | N | 79 | 77 | | |
| Ratio SAM/SAH T0 | Correlation Coefficient | ,077 | -0.047 | | |
|  | Sig. (2-tailed) | ,501 | 0.684 | | |
|  | N | 79 | 77 | | |
| Delta SAM T1-T0 | Correlation Coefficient | -,025 | 0.009 | | |
|  | Sig. (2-tailed) | ,832 | 0.936 | | |
|  | N | 77 | 75 | | |
| Delta SAH T1-T0 | Correlation Coefficient | ,022 | 0.074 | | |
|  | Sig. (2-tailed) | ,852 | 0.529 | | |
|  | N | 77 | 75 | | |
| Delta Ratio SAM/SAH T1-T0 | Correlation Coefficient | -,008 | -0.063 | | |
|  | Sig. (2-tailed) | ,942 | 0.591 | | |
|  | N | 77 | 75 | | |

* Spearman’s Rho Correlation Coefficients
