# Supplementary material for: Global methylation in relation to methotrexate-induced oral mucositis in children with acute lymphoblastic leukemia
Source: PLoS One. 2018 Jul 9;13(7):e0199574. doi: 10.1371/journal.pone.0199574 (PMC6037363; doi:10.1371/journal.pone.0199574)
Supplement: S5 Table — (DOCX) [file pone.0199574.s006.docx]

***Supplemental Table 4. LINE1 DNA methylation status (percentage) at T0 in relation to MTX-induced oral mucositis per single CpG site***

| Global *LINE1* DNA methylation | *No Mucositis n (%) Mucositis n (%)* | *Percentage methylation* | *p-value* |
| --- | --- | --- | --- |
| LINE1 - CpG1, mean *± SD* | 63 (79)  17 (21) | 64.6 ± 4.2 65.2 ± 3.6 | *0.603* |
| LINE1 - CpG2, mean *± SD* | 65 (79)  17 (21) | 59.2 ± 1.9 59.9 ± 1.4 | *0.160* |
| LINE1 - CpG3, mean *± SD* | 65 (79)  17 (21) | 70.5 ± 2.0 71.2 ± 1.9 | *0.239* |
| LINE1 - CpG5, mean *± SD* | 65 (79)  17 (21) | 38.8 ± 1.6 39.1 ± 1.2 | *0.595* |
| LINE1 - CpG6.7, mean *± SD* | 63 (79)  16 (21) | 70.6 ± 2.6 70.8 ± 2.5 | *0.838* |
| LINE1 - CpG8.9, median (range) | 65 (79)  17 (21) | 68.0 (58.5 – 72.3) 68.3 (65.7 – 71.3) | *0.350* |
| LINE1 - CpG11.12, median (range) | 65 (79)  17 (21) | 83.7 (77.0 – 86.3) 84.0 (80.7 – 86.0) | *0.318* |

*Percentage methylation of individual CpG sites in LINE1 (%) in patients with and without MTX-induced oral mucositis; mean ± SD or median (IQR) based on normal distribution of data*
