# Supplementary material for: Global methylation in relation to methotrexate-induced oral mucositis in children with acute lymphoblastic leukemia
Source: PLoS One. 2018 Jul 9;13(7):e0199574. doi: 10.1371/journal.pone.0199574 (PMC6037363; doi:10.1371/journal.pone.0199574)
Supplement: S6 Table — (DOCX) [file pone.0199574.s007.docx]

***Supplemental Table 5. Change in LINE1 DNA methylation status (Delta T1 – T0 percentage) during MTX therapy in relation to MTX-induced oral mucositis per single CpG site***

| Delta T1-T0 global *LINE1* DNA methylation | *No Mucositis n (%) Mucositis n (%)* | *Change in methylation* | *p-value* |
| --- | --- | --- | --- |
| LINE1 - CpG1, mean ± SD | 61 (78)  17 (22) | 3.9 ± 3.4 3.3 ± 4.0 | *0.564* |
| LINE1 - CpG2, median (range) | 63 (79)  17 (21) | 1.3 (-1.0 – +6.3) 0.7 (-1.0 – +5.3) | *0.532* |
| LINE1 - CpG3, mean ± SD | 63 (79)  17 (21) | -0.9 ± 12.6  0.8 ± 1.4 | *0.573* |
| LINE1 - CpG5, mean ± SD | 61 (78)  17 (22) | 0.4 ± 1.2  0.4 ± 1.2 | *0.911* |
| LINE1 - CpG6.7, mean ± SD | 59 (80)  15 (20) | -1.9 ± 13.5   0.7 ± 1.9 | *0.458* |
| LINE1 - CpG8.9, median (range) | 63 (79)  17 (21) | 1. (-1.0 – +3.3)   0.3 (-1.7 – +3.3) | *0.255* |
| LINE1 - CpG11.12, median (range) | 65 (79)  17 (21) | 1.0 (-1.0 – +3.7) 0.3 (-1.7 – +3.8) | *0.037* |

*T0: before start MTX; T1: after stop MTX. The change in percentage methylation of individual CpG sites in LINE1 (%) between T0 and T1 in patients with and without MTX-induced oral mucositis; mean ± SD or median (IQR) based on normal distribution of data*
